# ECI biocommentary: Jason M. Nagata

**DOI:** 10.1038/s41390-022-02305-3

**Published:** 2022-09-23

**Authors:** Jason M. Nagata

**Affiliations:** Division of Adolescent and Young Adult Medicine, Department of Pediatrics, University of California, San Francisco, San Francisco, CA USA

I grew up in Southern California^1^ and discovered my calling to medicine through global health coursework as an undergraduate at the University of Pennsylvania. Inspired to address global hunger and malnutrition in accordance with the United Nations Millennium Development Goals, my early research focused on malnutrition in Guatemala, Kenya, and with the World Health Organization.^2^ Furthering my studies, I pursued a master’s degree in medical anthropology at the University of Oxford. When I began medical school at the University of California, San Francisco (UCSF), my advisor suggested that I shadow in the adolescent eating disorders clinic given my research interests in nutrition. I soon found that I loved working with teens and selected the eating disorders clinic for my continuity clinic. Over the course of the year, I cared for a teenage boy who struggled with an eating disorder. Barriers in his care made me aware of the significant gaps in research and the lack of guidance for boys and men with eating disorders. This motivated my research on eating disorders in boys and men during my pediatrics residency at Stanford and adolescent medicine fellowship at UCSF. I am fortunate to stay at UCSF as faculty, which allows me to continue my research and clinical work in adolescent eating disorders.

**Figure:**
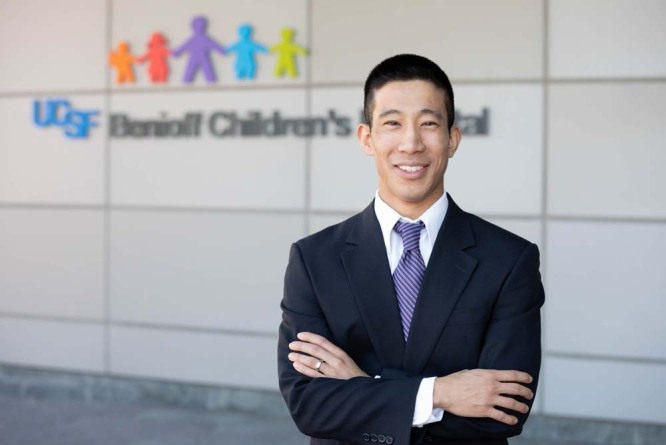
Susan Merrell

During the COVID-19 pandemic, our program experienced more than a doubling in hospitalizations for eating disorders. Screen time concurrently doubled given social distancing and the cancellation of events. Many of our patients expressed how increased social media use exacerbated their eating disorder symptoms. Because of this relationship, I became motivated to further investigate problematic social media and mobile phone use, which is the focus of the current study.^3^

Based on my experiences, there are two lessons I would like to pass on to future physician scientists. First, align your clinical work with your research when possible. Let your patients inspire you—your direct experiences caring for them will allow you to identify gaps and challenges in the field, serving as guidance for further research. Second, my mentors encouraged me to dream big and prioritize important research questions that could revolutionize fields or improve clinical management. This has led to my overall research objective to inform gaps in clinical and public health guidelines for adolescent and young adult health, including eating disorders in boys and men, as well as guidance for adolescent digital technology use.
